# Prevalence and incidence of neurological disorders among adult Ugandans in rural and urban Mukono district; a cross-sectional study

**DOI:** 10.1186/s12883-016-0732-y

**Published:** 2016-11-17

**Authors:** Mark Kaddumukasa, Leviticus Mugenyi, Martin N. Kaddumukasa, Edward Ddumba, Michael Devereaux, Anthony Furlan, Martha Sajatovic, Elly Katabira

**Affiliations:** 1 Department of Medicine, School of Medicine, Makerere University College of Health Sciences, P.O. Box 7072, Kampala, Uganda; 2 Infectious Diseases Research Collaboration, Mulago Hill Road, MUJHU3 Building, P.O. Box 7475, Kampala, Uganda; 3 Interuniversity Institute for Biostatistics and Statistical Bioinformatics, Hasselt University, Diepenbeek, Belgium; 4 Department of Medicine, St Raphael of St Francis Nsambya Hospital, Nkozi University, P.O. Box 7146, Kampala, Uganda; 5 University Hospitals Case Medical Center, Neurological Institute Case Western Reserve University, 11100 Euclid Avenue, 44106 Cleveland, OH USA; 6 Neurological and Behavioral Outcomes Center, University Hospitals Case Medical Center, 11100 Euclid Avenue, 44106 Cleveland, OH USA

**Keywords:** Epidemiology, Neurological disorders, Prevalence, Uganda

## Abstract

**Background:**

The burden of neurological diseases is increasing in developing countries. However, there is a prominent scarcity of literature on the incidence of neurological diseases in sub-Saharan Africa. This study was therefore undertaken to determine the prevalence and incidence of neurological diseases in this setting to serve as a baseline for planning and care for neurological disorders in Uganda.

**Methods:**

The study was conducted within rural and urban Mukono district, east of Kampala city of Uganda, central region. Over a period of six months, a cross sectional survey was conducted and screening was performed using a standardized questionnaire. All subjects with neurological symptoms and signs were reviewed by a team of neurologists and neurological diagnoses made.

**Results:**

Of the 3000 study subjects, 50.3% (1510/3000) were from the rural setting. Out of the participants screened, 67.4% were female, with a median age of 33 years. Among the 98 subjects with confirmed neurological disorders, the frequency of diseases was as follows; peripheral neuropathy (46.2%), chronic headaches (26.4%), and epilepsy (8.5%), followed by pain syndromes (7.5%), stroke (6.6%) and tremors/Parkinson disease (3.8%). The crude prevalence rates of these disorders (95% CI) were 14.3% (8.5–24.1); 13.3% (7.7–22.8); 33.7% (23.9–47.4) for stroke, epilepsy and peripheral neuropathy respectively. Peripheral neuropathy followed by chronic headaches had the highest estimated incidence/1000 years. Stroke had an estimated incidence of 3.6 new cases with 95% CI of (2.1–6.1)/1000 years.

**Conclusion:**

Peripheral neuropathy, chronic headaches and epilepsy disorders are major causes of morbidity in Sub-Saharan settings. There is an urgent need of more robust and powered studies to determine the incidence of these diseases.

**Electronic supplementary material:**

The online version of this article (doi:10.1186/s12883-016-0732-y) contains supplementary material, which is available to authorized users.

## Background

Neurological diseases contribute significantly to morbidity and mortality worldwide. Globally, stroke is the second leading cause of death after ischemic heart disease [1, 2]. The burden of neurological conditions in sub-Saharan Africa has been increasing over the years [3]. Neurological disorders contributed to 92 million disability-adjusted life-years (DALY) in 2005 and were projected to 103 million in 2030 worldwide [4]. The annual incidence of stroke has been estimated to be 316 per 100 000, and a prevalence of up to 315 per 100 000 with a three-year fatality of up to 84% in Africa [5]. The yearly age-adjusted rates of stroke per 100,000 people in the developing countries, the 15 to 64 age group averaged 49 per 100,000, four times the rates in developed countries. To date, most data on mortality have been hospital-based, although the majority of stroke deaths in the region are thought to occur at home [6].

Epidemiological data plays an important role in identification of disease occurrence and patterns as well as associated risk factors and etiology [7, 8]. The World Health Organization estimates that by the year 2030, 80% of all strokes will occur in low and middle income countries like Uganda [9] which are still battling with the scourge of communicable diseases like HIV/AIDS, malaria and tuberculosis. It is also clear that, as economies improve in resource-limited areas of the world, with associated changes in lifestyle, diet and environment, diseases more often associated with the Western world (such as stroke) are on the rise.

Neurological diseases may cause disproportionate burden in sub-Saharan Africa compared to developed countries. In Africa, stroke occurs in younger age-groups (30–69 years), and the resultant economic impact is felt on country’s health system as well as the loss of income and production of those affected either directly or indirectly by the disease [10]. Epilepsy is a much more frequent cause of death in Sub-Saharan Africa than in developed countries [11]. A community-based study done in Ethiopia revealed that 6.3% of people with epilepsy had died over a two-year period and one-third in 20 years [12]. In Africa, epilepsy mortality is primarily due to poorly controlled seizures and from accidents in addition to the sudden unexpected death in epilepsy (SUDEP) that is known throughout the world [11]. It is worth noting that these frequencies are likely underestimates, particularly in resource-limited areas of the world because of poor health reporting and limited health infrastructure. In spite of many Africans dying prematurely from neurological diseases, an epidemiological transition is being observed with an increase in longevity and increase in those who develop neurological diseases that more commonly present in later-life [13]. This transition and an increasing prevalence of neurological diseases in sub-Saharan Africa pose a major threat to livelihood and economic development.

Unfortunately, there is little epidemiological data in neurological conditions in many Sub-Saharan African countries. No adequate research has been done to determine the prevalence or incidence of neurological diseases within the Ugandan population. Therefore, our study objective was to determine the prevalence of neurological diseases in urban and rural populations of Mukono, a region surrounding the Ugandan capital of Kampala. This study serves as a starting point to inform policy on the prevalence and incidence of neurological diseases, and also serve as a baseline for planning and care for neurological disorders in Uganda.

## Methods

### Study design

This was a cross-sectional study.

### Study site and settings

The study was conducted in Mukono district which lies in the central region of Uganda. It shares boundaries with the districts of Buikwe in the East; Kayunga along River Sezibwa in the North; Luwero in the North West; Kampala and Wakiso in the South-West; and Lake Victoria and Tanzania in the South. The district headquarters are situated in Mukono town, 21 kms East of Kampala city. The 2002 population census projected Mukono’s population to be 536,400 people with a population density of 495 people per km^2^. Currently, the population under 18 years is 443,946; the youth (18–30) are 175,708, while those aged 60 years and above are 38,975 [14]. Mukono district has two health centre IV, 13 health centre III, 21 health centre II, 85 private clinics, 320 registered drug shops, 25 domiciliary maternity units and two private not for profit religious based hospitals.

Using a cross sectional community based survey, we randomly selected 3000 adults, aiming at interviewing at least one adult per household selected. About 1500 subjects from two sub-counties of Mukono Town Council and Kojja over a 6 months period from June 2014 – December 2014 were enrolled for this study. Mukono Town Council and Kojja sub-counties represented urban and rural populations respectively. This classification was based on information from the Mukono district administration and the current level of urbanization. During this community survey, 3000 adults were screened from the list of households randomly generated from a census database at each sub – county. The stepwise approach was followed as shown below; Administration of a questionnaire, physical measurements such as weight, height, waist hip ratio, blood pressure and neurological examination and laboratory measurements; including fasting blood glucose and lipid parameters [15].

### Sample size calculations

We considered a sample of 3000 participants, larger than 2232 which was used by Dewhurst et al. (2012) [16]. A larger sample size was considered to account for uncertainty of non-response.

We therefore selected 3000 study participants (1500 rural and 1500 urban) for this survey using multi-stage sampling procedure at the sub-county, parish and village level, using the sampling frame will be acquired from the enumeration study.

### Study procedures

#### Participant notification

A village was targeted for recruitment once their local council agreed to participate and community meetings explaining study activities had taken place. The next step of securing engagement with the study involved direct participant candidate notification, which happened a week before arrival of the survey teams. The notification procedures were carried out to ensure maximum participant availability when the survey teams arrived, and also provide an opportunity to answer any questions about the study.

Written informed consent from all study participants was obtained before enrolment, including the use of reliable intermediaries as appropriate to ensure that the implications of participation were fully understood. Eligible adult participants and emancipated minor participants also provided informed consent. Emancipated minors are defined by the Ugandan National guidelines for Research involving human as Research participants as individuals below the age of majority, who are pregnant, married, have a child or provide for their own livelihood [17].

### Participant recruitment

Our study used the modified version of a survey questionnaire developed by the World Health Organization (WHO) protocol for Epidemiological Neurological Disorders in developing countries which has been used by earlier studies [16, 18]. The sensitivity of the screening instrument ranges from 96% and specificity was 86% [19]. The interviewers were trained by neurologists in the research team.

### Survey screening (study phase I)

At the community level, house-to- house screening of residents was undertaken by trained interviewers. During the survey, only residential buildings were surveyed and demographic plus other relevant clinical information were obtained from the participants or household member such as a parent or first degree relative, if the person was unavailable or unable to respond appropriately to the questions asked.

**Fig. 1:**
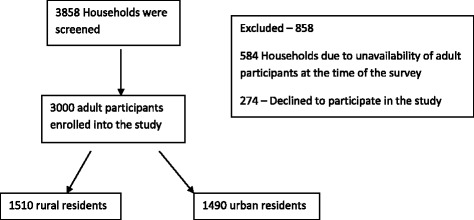
Showing the study flow diagram

All adult members of a household were eligible to take part in the survey, however only one adult member of the household member was selected to participate in the survey. If he/she agreed in principle to take part in the survey, a written informed consent was then obtained and the participant given a study number. The selected household member then would be requested to respond to the survey questionnaire. If an individual declined to participate then they would be replaced by other eligible household member where possible or the next household is approached (Fig. 1).

### Neurological examination (study phase II)

After the survey, study participants who were responded affirmatively for any neurological disorder in the screening form were scheduled for a complete neurological examination performed by a neurologist on a given day at the local health center nearby. A clinical examination questionnaire included additional pain, neuropathy, epilepsy, headache, Parkinson’s disease and stroke-specific questions to further validate the diagnosis. Disease condition duration, symptoms, drug history and risk factors were also assessed.

### Diagnostic criteria

The burden of the neurological disorders was determined as point prevalence, and defined as the proportion of patients who had a specific neurological diagnosis at the day of contact with the study researchers. The diagnoses were based on the WHO classifications of diseases [20]. The diagnoses of these diseases were considered definite if (1) Other physicians had previously the neurological condition and the study neurologist concurred with the diagnosis and (2) the study neurologist found presenting sequelae consistent with such a diagnosis.

### Collection of survey questionnaire data

After obtaining informed consent, the survey team collected the required information using a structured pre-tested questionnaire. The questionnaire was administered by face to face personal interviews at a research clinic or selected area, ensuring a setting that provided maximum privacy to conduct the interview, demographic data (age, sex, address), dietary, tobacco and alcohol consumption and medical history, socioeconomic status, family history and symptoms of neurological disease including chronic headaches, seizures, limb weakness etc. were assessed.

### Neurological assessments

The screening instrument for neurological disorders was adopted from a previous survey conducted in Tanzania having a sensitivity of 87.8% and specificity of 94.9% [16]. For all subjects who responded yes to the survey screening questionnaire an appointment for a neurological exam was scheduled within two days (Additional file 1). A full neurological history and examination was performed by a team of neurologists who would confirm or exclude a neurological disease. The individual results of the screening questionnaire, neurological history and examination were validated with a neurologist (KM, MNK, ED) were appropriate.

### Ethical considerations

Ethical approval for the study was obtained from Makerere University College of Health Sciences’ School of Medicine review board and ethics committee Ref number 2013–145 and Uganda National Council of Science and Technology Ref Number. HS1551. Written informed consent was obtained before enrolling the participants into the study.

### Statistical analysis

When dealing with binary outcomes from cross-sectional studies, several alternatives to logistic regression have been proposed to estimate prevalence, prevalence ratio, incidence rate and incidence rate ratio. Some of the alternative models include log-binomial regression, Poisson regression [21] and complementary log-log model, where the link function is log(− log(1 -π)) and the distribution is binomial [22]. In this paper we applied the Poisson model to estimate the prevalence and prevalence ratios, and complementary log-log model to estimate incidence and incidence rates. We fitted these models to each health condition (coded 0 if absent and 1 if present). We used only the intercept models to estimate both the prevalence and incidence rate. Later, we included covariates like sex into the models to estimate the ratios. For the complementary log-log model, the logarithm of the individual’s age (a) in year was set as an offset variable. The incidence of a particular condition was then estimated by exponentiation of the model coefficient. Since log(a), expressed as years of age, was included in the model as offset for each condition, the exponential of the constant in each case, is the estimated annual incidence rate. The estimates per 100,000 years rates were obtained simply by multiplying the annual incidence rates by 100,000. The latter rates were presented for comparison purposes with prior studies. All the analyses were done in STATA version 12 (Stata Corporation, College Station, TX, USA).

## Results

### Baseline demographics

A total of 3000 subjects were screened from 3858 households, with 50.3% (1510/3000) from the rural setting. Out of the participants screened, 67.4% were female, with a median age (IQR) of 33 (26–47) years. (See Table 1) Majority of the study participants 84.9% (2547/3000) were Baganda by tribe, which is the predominant tribe within the study settings.

**Table 1:** Distribution of the baseline characteristics of the screened patients

Baseline characteristic		Total	*N* (%)
Residence	Urban	3000	1490 (49.7)
	Rural	3000	1510 (50.3)
Gender	Male	2964	966 (32.6)
	Female	2964	1998 (67.4)
			Mean (SD)
Blood pressure: Systolic (A/)		2948	132.8 (22.3)
Blood pressure: Diastolic: (/B)		2948	81.0 (20.0)
Pulse		2947	80.2 (14.9)
Weight in Kg		2925	64.3 (14.9)
			Median (IQR)
Age in years		2936	33 (26–47)
Height in cm		2938	161.3 (156–167)
Body Mass Index (BMI)		2915	23.4 (20.8 – 27.4)
BMI classifications			
Under weight		2915	218 (7.5)
Normal weight		2915	1555 (53.3)
Over weight		2915	702 (24.1)
Obese		2915	440 (15.1)

### Response to the screening questionnaire

Among the study participants, 45.1% (1352/3000) responded yes to one or more of the study questions in the screening questionnaire. The most common response was having persistent loss of sensation (numbness) in arms or legs or hands or feet not due to cold weather 66.2% (895/1352). The least common response was having polio that resulted in long term problems/disability contributing 1.0% (14/1352). See Table 2.

**Table 2:** Distribution of the screened patients who responded yes to one or more of the study questions in the screening questionnaire

(*N* = 1352)	Yes N (%)
Have Persistent loss of sensation (numbness) in arms or legs or hands or feet that is not just due to cold or pain	895 (66.2)
Have severe recurrent headaches that stop one from doing normal daily activities	434 (32.1)
Have persistent weakness in any arms or legs, one side or both sides of the face	301 (22.3)
Have current weakness down one side of the body	267 (19.8)
Have problem opening or closing eye (s) that cannot be explained by tiredness or any other reason.	203 (15.0)
Have problem balancing on legs or difficulty walking that is due to pain or injury	163 (12.1)
Have recurrent attacks of violent shaking in one arm and/or one leg and/or in one side of the face that are not just brief tremors of the hands after working hard	108 (8.0)
Have arms or legs shake multiple times every day not due to alcohol or working hard	88 (6.5)
Have recurrent attacks involving falling with loss of consciousness and violent shaking of the limbs	87 (6.4)
Have shaking of hand, leg or trunk which cannot be controlled	85 (6.3)
Have neurological disorder (a disorder affecting brain, spinal cord or nerves)	68 (5.0)
Shuffles feet and takes small steps when walking not just because of pain	58 (4.3)
Have persistent weakness of one or both sides of face present	49 (3.6)
Ever had a stroke that resulted in long term problems/disability	47 (3.5)
Walk like one who has taken alcohol yet not	46 (3.4)
Have recurrent short attacks of severe shock like pain affecting one side of the face	40 (3.0)
Have persistent problems pronouncing words not just because of having a sore throat or mouth that affects him/her most of the time	36 (2.7)
Have persistent problems with coordination such as stirring a cup of tea with a spoon or buttoning clothes that is not due to pain in hands or arms or because of drinking alcohol	25 (1.9)
Have head shake all or most of the time such that it is visible	21 (1.6)
Have been told to have epilepsy or epileptic fits or seizures	21 (1.6)
Have been told to have Parkinson’s disease	19 (1.4)
Have ever had polio that resulted in long term problems/disability	14 (1.0)

Among the 1352 study participants, 5.0% (68) reported that they had been told that they have a neurological disorder. However, only 38.2% (26/68) could specify the type of the neurological disorder and of these 22.2% (15/68) noted that the neurological disorder was present at the time of the survey. Stroke and epilepsy were the most common accounting for 40 and 31.3% respectively. Other disease conditions specified included spinal cord problems, mental disorder and cerebral palsy with 2 (13.3), 1 (6.7) and 1 (6.7%) respectively.

### Prevalence of neurological disorders among the study participants

The highest prevalence of diagnoses made within the study group in descending order were; Peripheral neuropathy, chronic headaches, stroke epilepsy, pain syndromes and tremors (Table 3). Out of the 1352 respondents to the screening questionnaire, a total of 98 neurological cases were observed in this group of participants who turned up at the health facilities on the scheduled days. Peripheral neuropathy had 33 cases, chronic headaches 20 cases, stroke 14 cases, epilepsy 13 cases, pain syndromes 10 and tremors had 8 cases. Among the chronic headaches, cluster headaches had the lowest crude prevalence rate of 1% with 95% CI of 0.1–7.2.

**Table 3:** Crude prevalence of diagnosed diseases among the study population (*N* = 98)

Diagnosed diseases	Number of observed cases	Crude prevalence % (95% CI)
Stroke	14	14.3 (8.5–24.1)
Epilepsy	13	13.3 (7.7–22.8)
Pain syndrome	10	10.2 (5.5–19.0)
Peripheral neuropathy	33	33.7 (23.9–47.4)
Tremors	8	8.2 (4.1–16.3)
Chronic headaches	20	20.4 (13.2–31.6)

### Distribution of neurological diseases according to age and sex among the study participants

Nearly, two thirds of the study participants were female, 77.6% (76/98) and overall they were diagnosed with more diseases than males. Among those diagnosed with the following neurological disorders; pain syndromes, peripheral neuropathy, epilepsy, chronic headaches, and stroke, 70, 78.8, 69.2, 80 and 85.7% respectively were female. Migraine headaches were the most common type of chronic headache contributing 65%, with 85% of the sufferers being female. Stroke was more prevalent among females with prevalence of 15.8% compared to 9.1% for males. Females also had a higher prevalence (95% CI) of peripheral neuropathy 34.2% (23.3–50.2) and migraine headaches 14.5% (8.0–26.1) compared to males with 31.8% (15.2–66.7) and 9.1% (2.2–36.3) respectively. Male had a higher prevalence for epilepsy, pain syndromes, tremors and tension headaches (See Table 4).

**Table 4:** Prevalence of diagnosed diseases among the study population, adjusted for sex (male = 22, female = 76)

Diagnosed diseases	Sex	Number of observed cases	Prevalence % (95% CI)	Prevalence ratio (95% CI)
Stroke	Male	2	9.1 (2.3–36.3)	Reference
	Female	12	15.8 (9.0–27.8)	1.7 (0.4–7.8)
Epilepsy	Male	4	18.2 (6.8–48.4)	Reference
	Female	9	11.8 (6.2–22.8)	0.7 (0.2–2.1)
Pain syndrome	Male	3	13.6 (4.4–42.3)	Reference
	Female	7	9.2 (4.4–19.3)	0.7 (0.2–2.6)
Peripheral neuropathy	Male	7	31.8 (15.2–66.7)	Reference
	Female	26	34.2 (23.3–50.2)	1.1 (0.5–2.5)
Tremors	Male	2	9.1 (2.2–36.3)	Reference
	Female	6	7.9 (3.5–17.6)	0.9 (0.2–4.3)
Chronic headaches	Male	4	18.2 (6.8–48.4)	Reference
	Female	16	21.1 (12.9–34.4)	1.2 (0.4–3.5)

### Age distribution and estimated incidence according to the diseases

The subjects with epilepsy were younger with a median age in years (IQR) of 27 (22–32) compared to stroke subjects with median age of 57.5 (45–75) years. The median ages for peripheral neuropathy and pain syndromes were similar at 45 years. Among those with chronic headaches, the median age for migraine, tension and cluster was 40, 46.5 and 27 years respectively (Table 5).

**Table 5:** Median age for diagnosed diseases among the study population (*N* = 98)

Diagnosed diseases	Median age (IQR)
Stroke	57.5 (45–75)
Epilepsy	27 (22–32)
Pain syndrome	45 (27–60)
Peripheral neuropathy	45 (31–56)
Tremors	41.5 (27–65.5)
Chronic headaches	40.0 (26.5–52.5)

***un-estimable due to 1 case observed

Peripheral neuropathy followed by chronic headaches had the highest estimated incidence/1000 years. Stroke had an estimated incidence of 3.6 new cases with 95% CI of (2.1–6.1)/1000 years (Table 6). There were no differences among those who were normal, underweight, overweight and obese study participants (Table 7).

**Table 6:** Incidence of diagnosed diseases among the study population adjusted for age using log (age) as an offset variable (*N* = 98)

Diagnosed diseases	Incidence (95% CI) per	Incidence (95% CI) per
	1000 years^a^	100, 000 years^b^
Stroke	3.6 (2.1–6.1)	360 (210–610)
Epilepsy	3.2 (1.8–5.4)	320 (180–540)
Pain syndrome	2.4 (1.3–4.5)	240 (130–450)
Peripheral neuropathy	9.4 (6.6–13.2)	940 (660–1320)
Tremors	1.9 (0.9–3.9)	190 (90–390)
Chronic headaches	5.1 (3.3–8.0)	510 (330–800)

^a^Estimated number of new cases per 1000 years

^b^Estimated number of new cases per 100,000 years

**Table 7:** Prevalence of diagnosed diseases among the study population, adjusted for BMI (underweight = 11, normal = 49, over weight = 24, obese = 12)

Diagnosed diseases	MBI categories	Number observed cases	Prevalence % (95% CI)	Prevalence ratio 95% CI)
Stroke	Normal	7	14.3 (6.8–30.0)	Reference
	Under weight	1	9.1 (1.2–64.5)	0.6 (0.1–5.2)
	Over weight	2	8.3 (2.1–33.3)	0.6 (0.1–2.8)
	Obese	3	25.0 (8.1–77.5)	1.8 (0.5–6.8)
Epilepsy	Normal	7	14.3 (6.8–30.0)	Reference
	Under weight	1	9.1 (1.2–64.5)	0.6 (0.1–5.2)
	Over weight	5	20.8 (8.7–50.1)	1.5 (0.5–4.6)
	Obese	0	0	0
Pain syndrome	Normal	4	8.1 (3.1–21.8)	Reference
	Under weight	1	9.1 (1.2–64.5)	1.1 (0.1–10.0)
	Over weight	2	8.3 (2.1–33.3)	1.0 (0.2–5.6)
	Obese	3	25.0 (8.1–77.5)	3.1 (0.7–13.7)
Peripheral neuropathy	Normal	18	36.7 (23.1–58.3)	Reference
	Under weight	3	27.3 (8.8–84.6)	0.7 (0.2–2.5)
	Over weight	6	25.0 (11.2–55.6)	0.7 (0.3–1.7)
	Obese	5	41.7 (17.3–100)	1.1 (0.4–3.1)
Tremors	Normal	4	8.2 (3.1–21.8)	Reference
	Under weight	2	18.2 (4.5–72.7)	2.2 (0.4–12.2)
	Over weight	2	8.3 (2.1–33.3)	1.0 (0.2–5.6)
	Obese	0	0	0
Chronic headaches	Normal	6	18.4 (9.6–35.3)	Reference
	Under weight	1	27.3 (8.8–84.6)	1.5 (0.4–5.5)
	Over weight	5	29.2 (13.9–61.2)	1.6 (0.6–4.3)
	Obese	1	8.3 (1.2–59.2)	0.5 (0.1–3.6)

## Discussion

Sub-Saharan Africa is experiencing a dramatic increase in non-communicable diseases (NCDs) [23] including neurological conditions. Determining the distribution of neurological disorders in sub-Saharan Africa is needed to enable health care planning. There is a dearth of information on incidence and morbidity of non-communicable diseases in sub-Saharan Africa, including Uganda.

This is the first neurological disorders survey done in Uganda. The overall point prevalence of neurological diseases was 3.3%. The crude prevalence of stroke was 14.3%; (95% CI:8.5–24.1). This is within the worldwide prevalence of stroke which varies between 4 and 20 per 1000 population [24, 25]. The estimated incidence (95% CI) for stroke was 360 (210–610) per 100,000 years. This is higher than figures reported from Nigeria, 134/100,000 [26] but lower than settings such as 424/100,000 in Bombay, India, [27] 461/100,000 in Cotonou, Benin [28], and 620/100,000 in China [29]. Our observed trend of increasing stroke prevalence among women differs from earlier studies [25, 30, 31]. Plausible reasons for this observation include the fact that more women seek health care compared to men [32, 33]. Reports from hospital-based studies still show that more than half of stroke subjects admitted are female.

The crude prevalence of epilepsy in our survey was 13.3% (95% CI of 7.7–22.8) with an incidence rate of 320/100,000 years (95% CI of 180–540) in Mukono district. This is lower than rates reported in Egypt (550/100,000), UK (400/100,000) and India (883/100,000). However, this is higher than an earlier study conducted in Uganda which reported an overall crude incidence rate of 215 per 100000 person-years, (age-adjusted: 156 per 100000 person-years). People with epilepsy in our survey were not routinely attending and receiving anti-epileptic therapy for their seizure control. The treatment gap in our setting appears due to problems with medication access, lack of follow-up, and epilepsy stigma.

The overall prevalence of chronic primary headaches was 18.4% (95% CI), this is slightly lower than earlier studies in similar regions in Africa. A higher prevalence was recorded in Zambia (72%) (gender- and habitation-adjusted 61.6%) [34] whereas, lower prevalence was reported in Ethiopia (21.6%) [35] and Tanzania (23.1%) [36]. The differences in the prevalence could be attributed to different methodologies used, as well as cultural and population characteristics of the studied patients. Female predominance is an almost consistent finding in many other studies, which reflects the fact that primary headaches are more common in women [37].

Chronic headaches may remain under-detected by household members and even by general practitioners. There are little or no efforts to education communities on chronic headaches in our settings. However, the availability of pain relievers as over-the-counter medicines may be playing a role in reducing headache burden within communities. The majority of those diagnosed with chronic primary headaches had never sought formal medical care for their medical conditions.

Few studies have explored the presence of peripheral neuropathy within community populations. Majority of studies have explored neuropathy in subjects who are diabetic or HIV infected. Our study reports a crude prevalence (95% CI) of 33.7 (23.9–47.4). This was based on symptoms and clinical examination with a monofilament no objective assessment with electro-neuromyography was performed. This may over estimate the prevalence of peripheral neuropathy in our settings. Focused studies utilizing the electroneuromyography are needed to explore this. However, our prevalence rates are similar to the reported prevalence among HIV infected cohorts in a range of 35–52% [38–41]. In this study we did not exclude HIV infected individuals and this might have affected the results. Studies among HIV infected cohorts have reported a significant association of low serum albumin levels with presence of peripheral neuropathy symptoms in HIV-infected individuals [40, 42]. This study was conducted in rural and urban settings; it’s probable that low protein diet in our communities may have a role in this high prevalence and further studies are needed to explore this.

Majority of the study participants were Baganda by tribe, this is the predominant ethnic tribe in Uganda, though contributing about 17% of the total population of Uganda. Therefore these results may be interpreted with caution as they might not represent the true findings in other ethnic tribes within Uganda. National wide, multi-ethnic studies may be needed to address this.

However, there was a low participation of males in this study, probably because males have a poor health seeking behavior [43] and also being the main bread winners for their households might have been away during the time of the conduct of the survey.

### Strengths and limitations

The studied population is representative of the general population, including the rural and urban settings in Uganda. Other strengths include use of a standardised instrument to evaluate the presence of neurological disorders and neurological examination by a team of neurologists. Limitations include cross-sectional design and the fact that incidence is better estimated by prospective studies. Not all subjects who respondents to the study questionnaire followed-up to the health facilities for medical evaluation, this might have led to under-reporting of diseases. There was a low participation of males and this might not reflect a true prevalence among this population. The study enrolled only adults and excluded those aged less than 18 years, further studies are needed to describe the prevalence of neurological diseases in children.

## Conclusions

Neurological diseases identified in this large Ugandan survey are similar to those in the majority of tropical countries. Stroke, epilepsy, chronic headaches and peripheral nerve disorders were the most frequent neurological conditions. Further prospective studies need to be conducted to determine the true incidence of these diseases in our settings.

## Additional file

Additional file 1: Screening questions. (DOCX 157 kb)

## Abbreviations

MEPI: Medical education partnership initiative
TC: Town council

## References

[1] World Health Organization (2008). Mortality estimates by cause, age, and sex for the year 2008.

[2] Alwan AD, Galea G, Stuckler D. Development at risk: addressing noncommunicable diseases at the United Nations high-level meeting. Bull World Health Organ. 89 (8):546-546A. 10.2471/BLT.11.091074 PMC3150770 21836748

[3] Lopez AD, Mathers CD, Ezzati M, Jamison DT, Murray CJL (2006). Measuring the global burden of disease and risk factors, 1990–2001.

[4] World Health Organisation. Neurological Disorders: Public Health Challenges. Geneva: World Health Organisation; 2006. http://www.who.int/mental_health/neurology/neurodiso/en/. Accessed 12 Nov 2015.

[5] Owolabi MO. Taming the burgeoning stroke epidemic in Africa: stroke quadrangle to the rescue. West Indian Med J. 2011;60(4):412–421. 22097671

[6] Kahn K, Tollman SM (1999). Stroke in rural South Africa--contributing to the little known about a big problem. S Afr Med J.

[7] Kurtzke JF (1984). Neuroepidemiology. Ann Neurol.

[8] Cockerell OC, Sander JW, Shorvon SD (1993). Neuroepidemiology in the United Kingdom. J Neurol Neurosurg Psychiatry.

[9] Mathers CD, Loncar D, Organisation World Health (2005). Updated projections of global mortality and burden of disease, 2002–2030: data sources, methods and results. Evidence and information for policy working paper.

[10] O'Donnell MJ, Xavier D, Liu L, Zhang H, Chin SL, Rao-Melacini P, Rangarajan S, Islam S, Pais P, McQueen MJ et al. Risk factors for ischaemic and intracerebral haemorrhagic stroke in 22 countries (the INTERSTROKE study): a case-control study. Lancet. 376 (9735):112–123. 10.1016/S0140-6736(10)60834-3 20561675

[11] Silberberg D, Katabira E (2006). Neurological Disorders.

[12] Tekle-Haimanot R, Forsgren L, Ekstedt J (1997). Incidence of epilepsy in rural central Ethiopia. Epilepsia.

[13] World Health Organisation (2007). Neurological disorders: public health challenges.

[14] Uganda Bureau of Statistics (2002). Uganda population and housing census.

[15] The WHO recommended STEP-Wise approach (The WHO STEPwise approach to Surveillance of noncommunicable diseases (STEPS). [http://www.who.int/ncd_surveillance]. Accessed 15 Nov 2015.

[16] Dewhurst F, Dewhurst MJ, Gray WK, Aris E, Orega G, Howlett W, Warren N, Walker RW. The prevalence of neurological disorders in older people in Tanzania. Acta Neurol Scand. 2013;127(3):198–207. 10.1111/j.1600-0404.2012.01709.x 22845781

[17] Uganda National Council for Science and Technology (UNCST) (2014). National guidelines for research involving humans as research participants.

[18] World Health Organization (2012). WHO protocol: epidemiologic studies of neurologic disorders. Acta Neurol Scand.

[19] Meneghini F, Rocca WA, Grigoletto F, Morgante L, Reggio A, Savettieri G, Di Perri R, Anderson DW (1991). Door-to-door prevalence survey of neurological diseases in a sicilian population. Background and methods. The Sicilian Neuro-Epidemiologic Study (SNES) Group. Neuroepidemiology.

[20] WHO Library Cataloguing-in-Publication Data (2010). International statistical classification of diseases and related health problems, 10th revision, edition 2010.3 v. Contents: v. 1. Tabular list - v. 2. Instruction manual - v. 3. Alphabetical index. 1.Diseases - classification. 2.Classification. 3.Manuals.

[21] Traissac P, Martin-Prevel Y, Delpeuch F, Maire B (1999). Logistic regression vs other generalized linear models to estimate prevalence rate ratios. Rev Epidemiol Sante Publique.

[22] Martuzzi M, Elliott P (1998). Estimating the incidence rate ratio in cross-sectional studies using a simple alternative to logistic regression. Ann Epidemiol.

[23] Ahmad OBB-PC, Lopez AD, Murray CJ, Lozano R, Inoue M (2001). Age standardization of rates: a new WHO standard.

[24] WHO MONICA Project Investigators (1988). The World Health Organization MONICA Project (Monitoring trends and determinants in cardiovascular disease). J Clin Epidemiol.

[25] Feigin VL, Lawes CM, Bennett DA, Anderson CS (2003). Stroke epidemiology: a review of population-based studies of incidence, prevalence, and case-fatality in the late 20th century. Lancet Neurol.

[26] Sanya EO, Desalu OO, Adepoju F, Aderibigbe SA, Shittu A, Olaosebikan O. Prevalence of stroke in three semi-urban communities in middle-belt region of Nigeria: a door to door survey. Pan Afr Med J. 2015;20:33. 10.11604/pamj.2015.20.33.4594 PMC4441143 26029322

[27] Bharucha NE, Bharucha EP, Bharucha AE, Bhise AV, Schoenberg BS (1988). Prevalence of stroke in the Parsi community of Bombay. Stroke.

[28] Cossi MJ, Gobron C, Preux PM, Niama D, Chabriat H, Houinato D. Stroke: prevalence and disability in Cotonou, Benin. Cerebrovasc Dis. 2012;33(2):166–172. 10.1159/000334195 22222467

[29] Li SC, Schoenberg BS, Wang CC, Cheng XM, Bolis CL, Wang KJ (1985). Cerebrovascular disease in the People’s Republic of China: epidemiologic and clinical features. Neurology.

[30] Osuntokun BO, Bademosi O, Akinkugbe OO, Oyediran AB, Carlisle R (1979). Incidence of stroke in an African City: results from the stroke registry at Ibadan, Nigeria, 1973–1975. Stroke.

[31] Ogun SA, Ojini FI, Ogungbo B, Kolapo KO, Danesi MA (2005). Stroke in south west Nigeria: a 10-year review. Stroke.

[32] Vlassoff C (2007). Gender differences in determinants and consequences of health and illness. J Health Popul Nutr.

[33] Buvinic M, Medici A, Fernandez E, Torres AC (2006). Gender Differentials in Health.

[34] Mbewe E, Zairemthiama P, Yeh HH, Paul R, Birbeck GL, Steiner TJ (2015). The epidemiology of primary headache disorders in Zambia: a population-based door-to-door survey. J Headache Pain.

[35] Mengistu G, Alemayehu S (2013). Prevalence and burden of primary headache disorders among a local community in Addis Ababa, Ethiopia. J Headache Pain.

[36] Dent W, Spiss H, Helbok R, Matuja W, Scheunemann S, Schmutzhard E (2004). Prevalence of migraine in a rural area in South Tanzania: a door-to-door survey. Cephalalgia.

[37] Bahrami P, Zebardast H, Zibaei M, Mohammadzadeh M, Zabandan N (2012). Prevalence and characteristics of headache in Khoramabad, Iran. Pain Physician.

[38] Keswani SC, Pardo CA, Cherry CL, Hoke A, McArthur JC (2002). HIV-associated sensory neuropathies. AIDS.

[39] Maritz J, Benatar M, Dave JA, Harrison TB, Badri M, Levitt NS, Heckmann JM (2010). HIV neuropathy in South Africans: frequency, characteristics, and risk factors. Muscle Nerve.

[40] Tumusiime DK, Musabeyezu E, Mutimurah E, Hoover DR, Shi Q, Rudakemwa E, Ndacyayisenga V, Dusingize JC, Sinayobye JD, Stewart A (2014). Over-reported peripheral neuropathy symptoms in a cohort of HIV infected and uninfected Rwandan women: the need for validated locally appropriate questionnaires. Afr Health Sci.

[41] Arenas-Pinto A, Thompson J, Musoro G, Musana H, Lugemwa A, Kambugu A, Mweemba A, Atwongyeire D, Thomason MJ, Walker AS et al. Peripheral neuropathy in HIV patients in sub-Saharan Africa failing first-line therapy and the response to second-line ART in the EARNEST trial. J Neurovirol. 2016;22(1):104–113. 10.1007/s13365-015-0374-7 26323809

[42] Dusingize JC, Hoover DR, Shi Q, Mutimura E, Kiefer E, Cohen M, Anastos K (2012). Association of serum albumin with markers of nutritional status among HIV-infected and uninfected Rwandan women. PLoS One.

[43] Tudiver F, Talbot Y (1999). Why don’t men seek help? Family physicians’ perspectives on help-seeking behavior in men. J Fam Pract.

