# Supplementary material for: Prevalence and incidence of neurological disorders among adult Ugandans in rural and urban Mukono district; a cross-sectional study
Source: BMC Neurol. 2016 Nov 17;16:227. doi: 10.1186/s12883-016-0732-y (PMC5114749; doi:10.1186/s12883-016-0732-y)
Supplement: Additional file 1: — Screening questions. (DOCX 157 kb) [file 12883_2016_732_MOESM1_ESM.docx]

**Screening questions**

| 1. Do you have recurrent attacks in which you fall with loss of consciousness and have violent shaking of the limbs? | YES NO |
| --- | --- |
| 1. Do you have recurrent attacks of violent shaking in one arm and/or one leg and/or in one side of the face that are not just brief tremors of the hands after working hard? | YES NO |
| 1. Do your arms or legs shake multiple times every day not just when you have drunk alcohol or have been working hard? | YES NO |
| 1. Do you shuffle your feet and take small steps when you walk not just because of pain? | YES NO   |
| 1. Does your head shake all or most of the time such that it is visible? | YES NO |
| 1. Do you have shaking of your hand, leg or trunk which you cannot control? | YES NO |
| 1. Do you have weakness down one side of your body that is there now? | YES NO |
| 1. Do you have persistent weakness in any of your arms or legs? Is it one side or both sides of the face? | YES NO |
| 1. Do you have persistent weakness of one or both sides of your face that is there now? | YES NO |
| 1. Do you have problem opening or closing your eye (s) that cannot be explained by tiredness or any other reason? When is it worst? | YES NO |
| 1. Do you have persistent problems pronouncing words not just because you have a sore throat or mouth and that affects you most of the time? | YES NO |
| 1. Do you have persistent loss of sensation (numbness) in your arms or legs or hands or feet that is there now and not just due to cold or pain? | YES NO |
| 1. Do you have persistent problems with your coordination such as stirring a cup of tea with a spoon or buttoning clothes that is not due to pain in your hands or arms or because of drinking alcohol? | YES NO |
| 1. Do you have recurrent short attacks of severe shock like pain affecting one side of your face? | YES NO |
| 1. Do you have severe recurrent headaches that stop you doing your normal daily activities? | YES NO |
| 1. Has anyone ever told you that you have a neurological disorder (a disorder affecting your brain, spinal cord or nerves)?   a) What is it  b) Is it still there? | YES NO |
| 1. Have you been told that you have epilepsy or epileptic fits or seizures? | YES NO |
| 1. Have you been told that you have Parkinson’s disease? | YES NO |
| 1. Have you ever had a stroke that resulted in long term problems/disability? | YES NO |
| 1. Have you ever had polio that resulted in long term problems/disability? | YES NO |
| 1. Do you have problem balancing on your legs or difficulty walking that is due to pain or injury? | YES NO |
| 1. Do you walk like you have taken alcohol yet you haven’t? | YES NO |

For question 16, if someone answered yes, a response of (a) was checked to see if it was a neurological disorder and a response of (b) was checked to ensure it was still there.
